# Cholecystogastric fistula presenting with transient gallstone ileus and incidental Fitz–Hugh–Curtis syndrome: A rare radiological and intraoperative association

**DOI:** 10.1016/j.radcr.2026.07.128

**Published:** 2026-08-30

**Authors:** Yigeng Li, Hasanga Jayasekera

**Affiliations:** Department of Surgery, Bendigo Health, Victoria, Australia

**Keywords:** Cholecystogastric fistula, Gallstone ileus, Fitz–Hugh–Curtis syndrome, Multimodal imaging

## Abstract

Cholecystogastric fistula (CGF) is the rarest subtype of cholecystoenteric fistula and is an uncommon complication of chronic gallstone disease. Diagnosis is often challenging because of nonspecific clinical features and subtle imaging findings. We report a rare case of CGF presenting with transient gallstone ileus and an incidental intraoperative finding of Fitz–Hugh–Curtis syndrome, a previously unreported association. A 42-year-old woman presented with acute epigastric pain and vomiting on a background of intermittent chronic epigastric discomfort. Contrast-enhanced computed tomography demonstrated an 18-mm lamellated gallstone within the distal ileum with mild proximal small bowel dilatation, suspicious for early gallstone ileus, together with features of chronic cholecystitis. Magnetic resonance cholangiopancreatography subsequently identified a 6mm fistulous tract between the gallbladder and gastric antrum, confirming a cholecystogastric fistula. Diagnostic laparoscopy confirmed the fistula, while no obstructing gallstone was identified, suggesting spontaneous passage into the colon. Characteristic perihepatic “violin-string” adhesions and pelvic adhesions consistent with Fitz–Hugh–Curtis syndrome were incidentally identified intraoperatively. The patient recovered uneventfully and was discharged on postoperative day 1. This case highlights the complementary roles of computed tomography (CT) and magnetic resonance cholangiopancreatography (MRCP) in diagnosing cholecystogastric fistula, with CT identifying indirect features of gallstone ileus and MRCP clearly delineating the fistulous communication. Recognition of transient gallstone ileus and awareness of the potential coexistence of Fitz–Hugh–Curtis syndrome may facilitate diagnosis, operative planning, and interpretation of complex hepatobiliary anatomy. To our knowledge, this represents the first reported association between cholecystogastric fistula and Fitz–Hugh–Curtis syndrome.

## Introduction

Cholecystoenteric fistula (CEF) is a rare complication of biliary tract disease, with cholecystogastric fistula (CGF) representing the rarest subtype [1]. Most cases arise secondary to chronic gallstone disease, where sustained inflammation, pressure necrosis, and gradual erosion of adjacent tissues result in fistula formation [[1], [2], [3]]. Although uncommon, persistent CGF may lead to serious complications, including gallstone ileus and Bouveret’s syndrome, and has also been associated with an increased incidence of gallbladder and gastric malignancy [4].

Clinical presentation is frequently nonspecific, commonly including epigastric or right hypochondriac pain, nausea, vomiting, fever, or jaundice, which contributes to delayed diagnosis [5,6]. Medical imaging including computed tomography (CT) and magnetic resonance cholangiopancreatography (MRCP) remains central to diagnosis and may demonstrate closely approximated edematous organs, pericholecystic inflammatory change, ectopic gallstones, and occasionally direct visualization of the fistulous tract [7]. Despite increasing recognition, optimal management remains debated, with multiple operative and nonoperative strategies described [8,9].

Fitz–Hugh–Curtis syndrome is characterized by perihepatic inflammation and “violin-string” adhesions, typically associated with pelvic inflammatory disease [10]. To our knowledge, there are no published reports describing coexistence of cholecystogastric fistula with Fitz–Hugh–Curtis syndrome. We present a rare case of CGF complicated by transient gallstone ileus with incidental intraoperative findings consistent with Fitz–Hugh–Curtis syndrome, highlighting a previously unreported association and its potential clinical implications.

## Case presentation

A 42-year-old woman presented to the emergency department with a 1-day history of acute epigastric pain associated with vomiting, on a background of intermittent chronic epigastric discomfort previously attributed to gastro-esophageal reflux disease and managed with proton pump inhibitors. She was sexually active with a single partner and reported no prior history of sexually transmitted infections. She had no significant past medical or surgical history.

On examination, the abdomen was soft with mild epigastric tenderness. There was no right upper quadrant tenderness and Murphy’s sign was negative. She was hemodynamically stable and afebrile.

Initial laboratory investigations demonstrated a mild leukocytosis (white cell count 11 × 10⁹/L) with minimally elevated C-reactive protein (3.1 mg/L). Liver biochemistry was deranged, with total bilirubin 26 µmol/L, alanine aminotransferase 162 U/L, aspartate aminotransferase 77 U/L, gamma-glutamyl transferase 248 U/L, and alkaline phosphatase 167 U/L. Serum lipase was normal (18 U/L). Urinalysis was positive for leukocytes, nitrites, and blood. Midstream urine culture subsequently grew *Escherichia coli*. Cervical screening performed during the admission was positive for non-16/18 high-risk human papillomavirus (HPV).

**Fig. 1:**
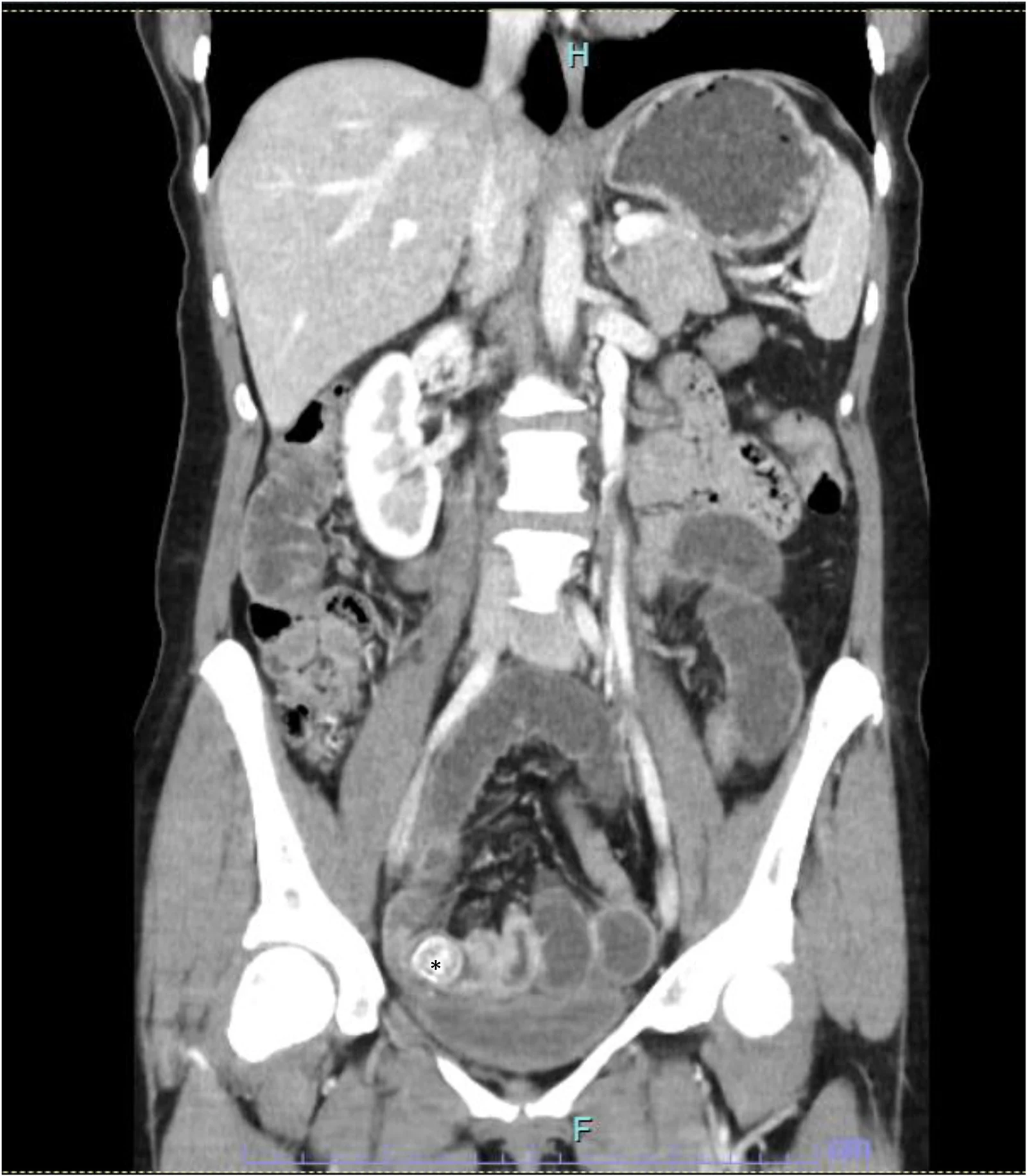
Hyperdense material in the right iliac fossa (asterisk) with proximal small bowel dilatation, suspicious for gallstone ileus.

Contrast-enhanced CT of the abdomen and pelvis demonstrated an 18-mm hyperdense lamellated gallstone within the distal ileum, associated with mild small bowel dilatation up to 3 cm, raising concern for early gallstone ileus (Fig. 1). The gallbladder appeared thick-walled and inflamed, containing multiple calculi.

**Fig. 2:**
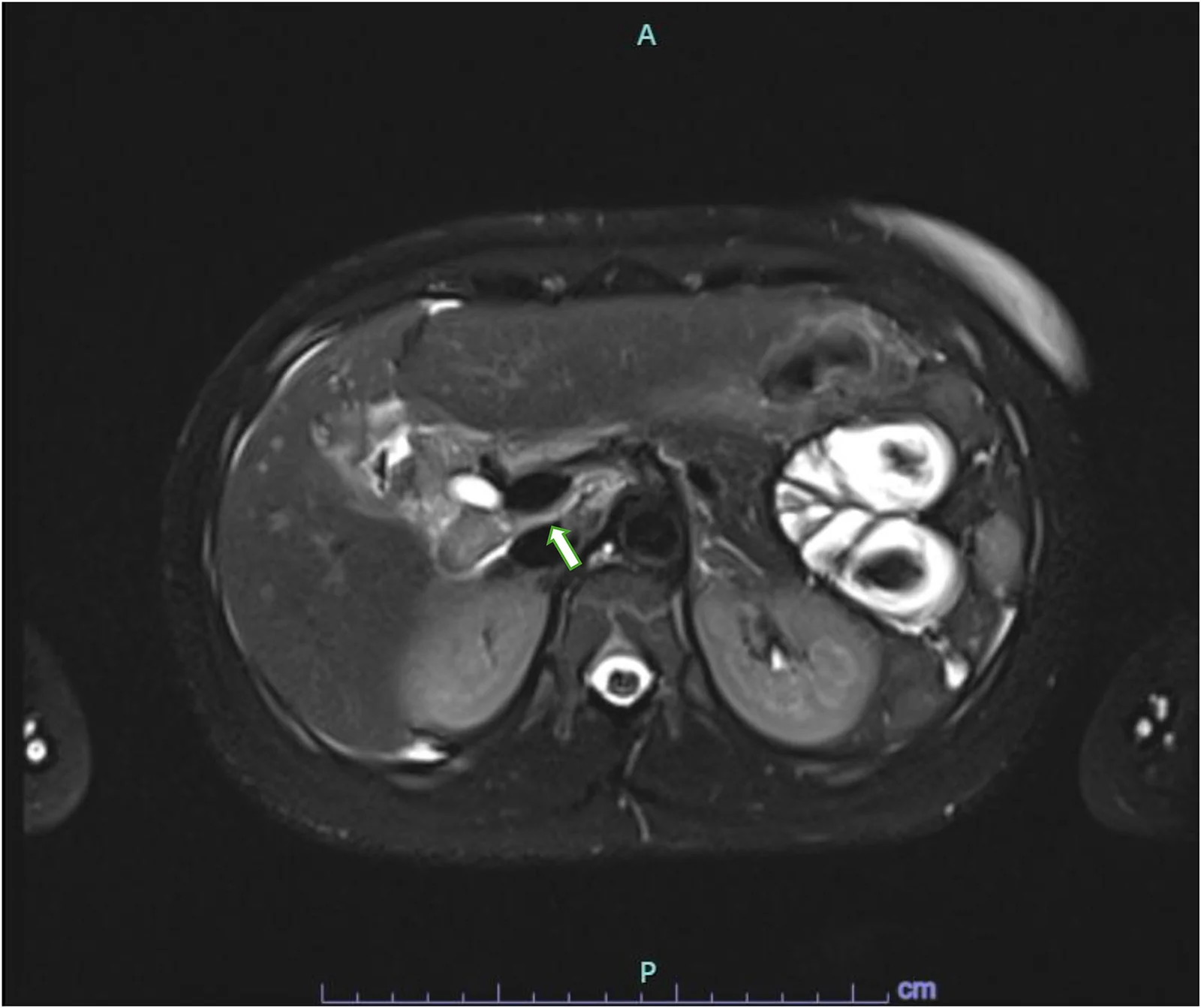
Axial T2 weighted image showing fistula (arrowhead) between the gallbladder and the gastric antrum.

**Fig. 3:**
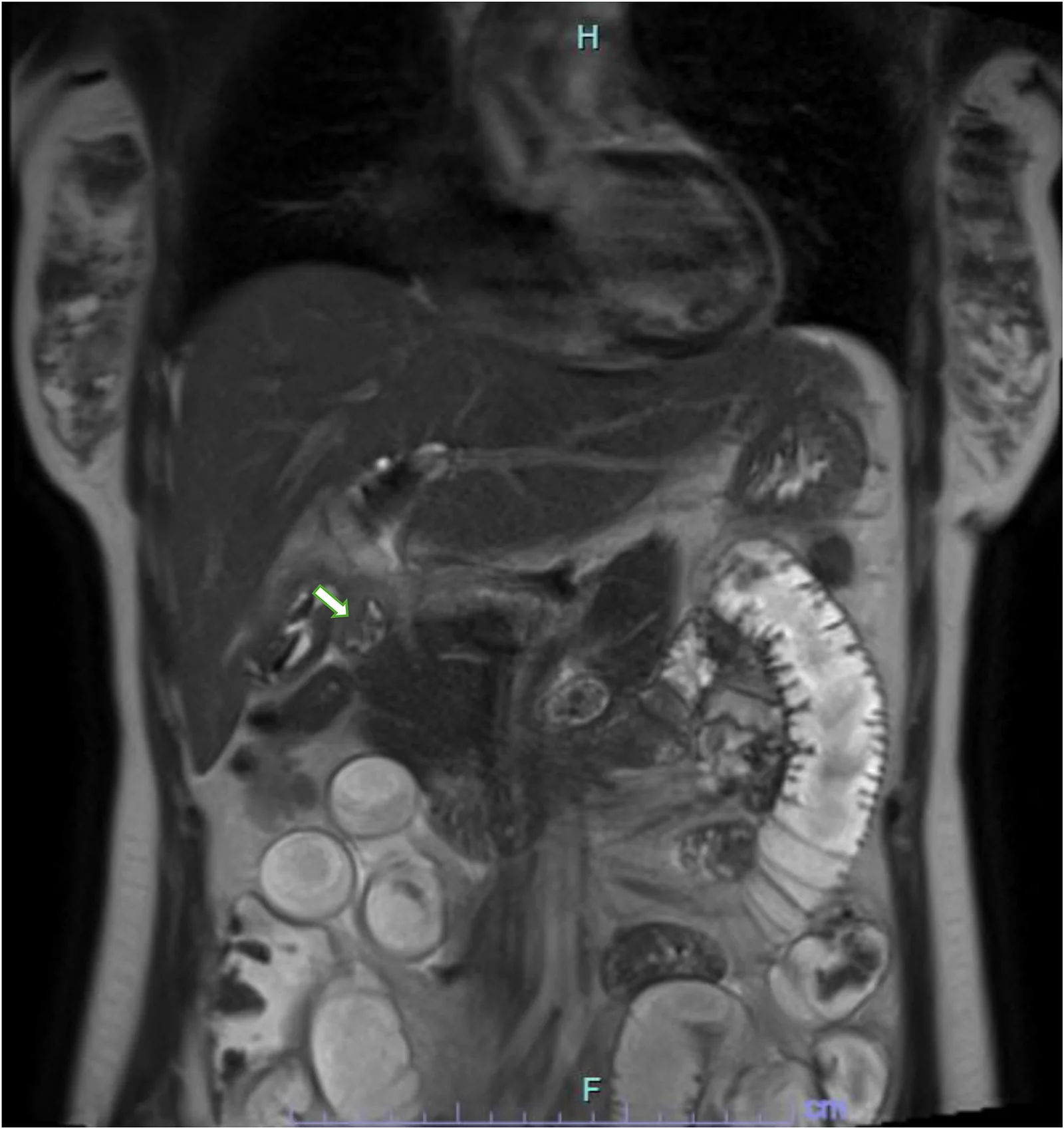
Coronal T2 weighted image showing the connection (arrowhead) between gallbladder and gastric antrum.

MRCP was completed on the next day which demonstrated the gallbladder was poorly distended with wall thickening, pericholecystic edema, and multiple calculi, consistent with chronic cholecystitis and fistulous communication measuring approximately 6 mm in diameter was identified between the gallbladder and the gastric antrum, confirming a cholecystogastric fistula (Fig. 2, Fig. 3).

**Fig. 4:**
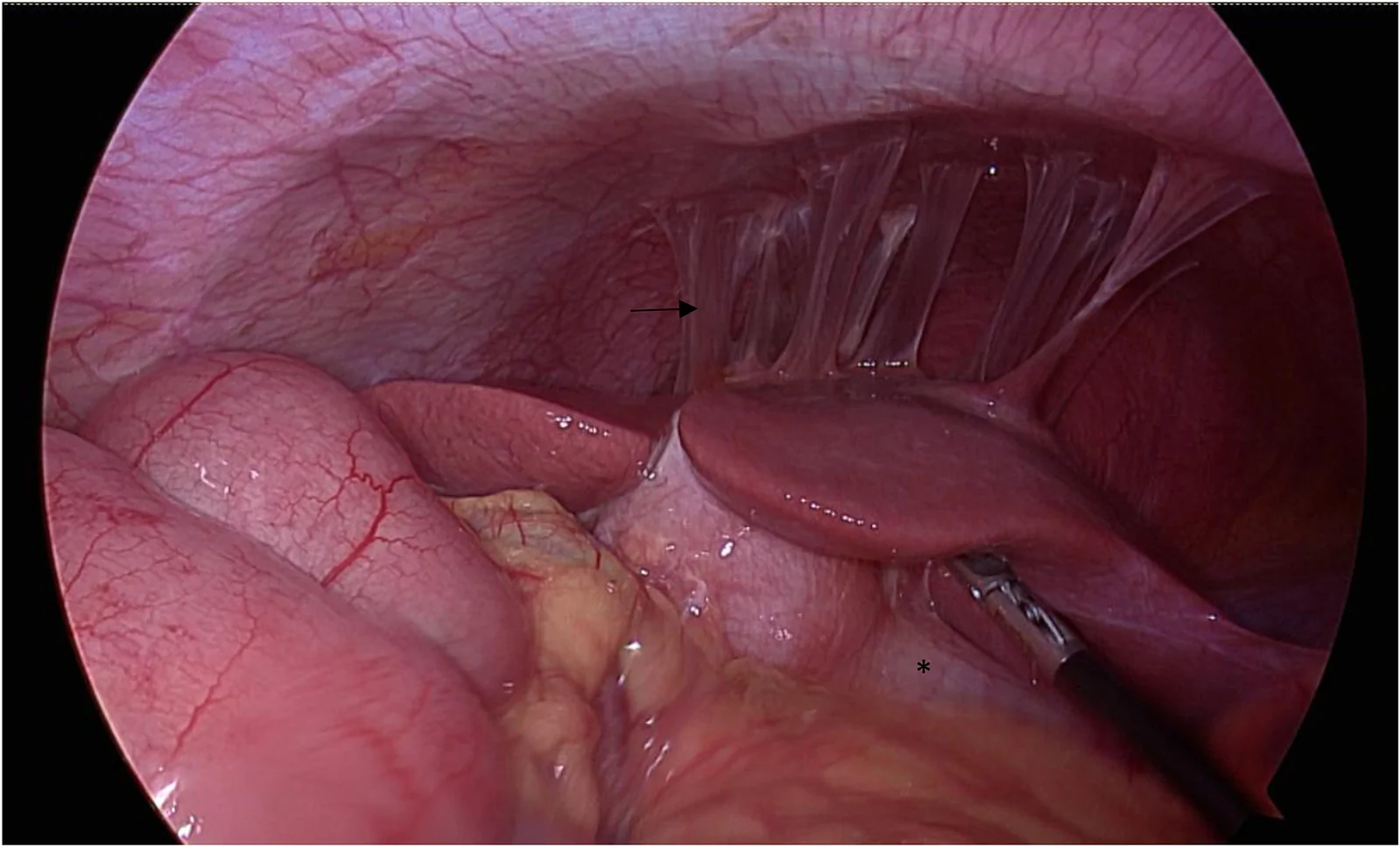
Intraoperative photo showing the cholecystogastric fistula (asterisk) and “violin-string” adhesions (arrow).

The patient was admitted under the general surgery service and underwent diagnostic laparoscopy on day 1 postadmission following MRCP. Intraoperatively, no gallstone was identified on running the bowel from the terminal ileum to the duodenojejunal flexure, suggesting spontaneous passage into the colon. A cholecystogastric fistula was confirmed. Adhesions were noted within the pelvis surrounding the uterus, as well as characteristic “violin-string” adhesions between the liver capsule and anterior abdominal wall (Fig. 4). An intraoperative gynecology consultation concluded that these findings were consistent with Fitz-Hugh–Curtis syndrome.

Postoperatively, the patient recovered uneventfully apart from a brief episode of hypotension. She was discharged on postoperative day 1 with plans for outpatient ultrasonography and endoscopy.

## Discussion

Cholecystogastric fistula (CGF) is the rarest subtype of cholecystoenteric fistula and remains diagnostically challenging due to its low prevalence and often subtle imaging findings [1]. The underlying mechanism involves chronic inflammation, pressure necrosis, and progressive erosion of the gallbladder into adjacent gastrointestinal structures, most commonly driven by gallstone disease [2,3], although alternative causes such as malignancy, infection, trauma, and peptic ulcer disease have been reported [11,12].

Medical imaging plays a central role in the evaluation of suspected biliary–enteric fistulas. Typical CT features include close approximation of the gallbladder and stomach with wall thickening, pericholecystic inflammatory change, pneumobilia, ectopic gallstones, and occasionally direct visualization of the fistulous tract [7]. Ancillary findings such as gas within the gallbladder adjacent to the stomach and focal gastric wall thickening further support the diagnosis [3].

In this case, CT demonstrated a hyperdense gallstone within the distal ileum with mild proximal small bowel dilatation, raising suspicion for early gallstone ileus. Subsequent MRCP confirmed a fistulous communication between the gallbladder and gastric antrum, illustrating the complementary role of MRCP in delineating biliary anatomy when CT findings are equivocal [13].

Gallstone migration through a fistula may result in obstruction at various gastrointestinal levels, including the ileocecal junction or proximal gastric outlet in Bouveret’s syndrome [[14], [15], [16]]. Risk factors include advanced age, female sex, gallstone diameter greater than 2.5 cm, and altered gastrointestinal anatomy [17,18]. Imaging plays a key role in localizing obstruction and guiding management, which may range from surgical to endoscopic or conservative approaches [8,9].

A unique feature of this case was the incidental intraoperative finding of perihepatic “violin-string” adhesions consistent with Fitz–Hugh–Curtis syndrome. To our knowledge, this association has not been previously reported. Although identified surgically, awareness of this coexistence may assist surgeons and radiologists in interpreting complex hepatobiliary anatomy and unexplained perihepatic inflammatory changes in similar presentations.

This case emphasizes the importance of multimodal imaging in diagnosing rare biliary–enteric fistulas and highlights a novel association with Fitz–Hugh–Curtis syndrome.

## Conclusion

Cholecystogastric fistula is a rare biliary–enteric complication that requires a high index of radiological suspicion. This case highlights the complementary roles of CT and MRCP in diagnosis, with CT identifying indirect features of gallstone ileus and MRCP clearly delineating the fistulous tract when CT findings are equivocal. The coexistence with Fitz–Hugh–Curtis syndrome represents a previously unreported association and expands the radiological context in which this entity may be encountered.

## Patient consent

The authors confirm that written informed consent was obtained from the patient for publication of this case report and any accompanying images.
